# A Transcriptomic Analysis Reveals Diverse Regulatory Networks That Respond to Cold Stress in Strawberry (*Fragaria*×*ananassa*)

**DOI:** 10.1155/2019/7106092

**Published:** 2019-08-05

**Authors:** Yong Zhang, Yunting Zhang, Yuanxiu Lin, Ya Luo, Xiaorong Wang, Qing Chen, Bo Sun, Yan Wang, Mengyao Li, Haoru Tang

**Affiliations:** ^1^College of Horticulture, Sichuan Agricultural University, Chengdu 611130, China; ^2^Institute of Pomology and Olericulture, Sichuan Agricultural University, 611130 Chengdu, China

## Abstract

Strawberry is often subjected to cold stress in temperate regions when insulation measures are not strictly applied in protected cultivation. Cold stress adversely influences plant growth and development by triggering a massive change to the transcriptome. To provide the potential strategies in improving strawberry cold tolerance and give a glimpse into the understanding of the complex cold signaling pathways in plants, this study identified attractive candidate genes and revealed diverse regulatory networks that responded to cold stress in strawberry (*Fragaria×ananassa*) by a transcriptomic analysis. Totally, there were 2397 differentially expressed genes (DEGs) under cold stress treatment (T1) *vs.* normal treatment (CK). Of these, 1180 DEGs were upregulated, while 1217 DEGs were downregulated. Functional enrichment analysis showed that DEGs were significantly (adjusted *P* value < 0.05) overrepresented in six pathways including plant hormone signal transduction, flavonoid biosynthesis, mitogen-activated protein kinase (MAPK) signaling, starch and sucrose metabolism, circadian rhythm, and alpha-linolenic acid metabolism. The cold signaling initiated expression of downstream cold-responsive (COR) genes with cis-acting element ABRE or CRT/DRE in the ABA-independent or ABA-dependent pathway to impel plant defense against the stress. Strikingly, GIGANTEA (gene id 101308922), two-component response regulator-like PRR95 (gene id 101295449), and ethylene-responsive transcription factor ERF105-like (gene id 101295082) were dramatically induced under low-temperature treatment, indicating that they played an important role in response to cold stress in strawberry.

## 1. Introduction

Owing to their sessile lifestyle, plants are forced to have evolved a variety of adaptive mechanisms to cope with the ever-changing environment and stress elicitors [1]. Cold stress, a low-temperature injury involving freezing (<0°C) and chilling (0-15°C), is one of the major environmental stress factors, which adversely affect plant growth and development and greatly limit agricultural productivity and geographical distribution [2]. Plant in acclimation to cold stress will trigger reorchestrating of metabolism, remodeling of cell and tissue structures, and reprogramming of gene expression [3, 4].

It has been extensively reported that cold stress triggers the increase of the endogenous ABA level and the exogenous application of ABA improves the cold tolerance of plants [5, 6]. The recent evidences suggest that the cold stress-induced change in plant growth and development is tightly linked to the intracellular IAA gradient [7]. Meanwhile, other hormones have been demonstrated to be involved in response to cold stress [8–11]. Collectively, the intricate interaction and crosstalk among various plant hormones control a wide range of physiological processes in mediating plant cold response. Additionally, sugar metabolism always allied with hormone signaling to regulate the growth, development, and stress response in plants [12, 13] and various sugars such as sucrose, trehalose, fructans, and raffinose participate in maintaining membrane integrity under cold stress [14].

Large-scale profiling of gene transcripts has revealed a sweeping change to the plant transcriptome, elucidating a diversity of transcriptional regulatory networks in response to the cold signal [2]. The CBF/DREB1- (C-repeat binding factor/dehydration-responsive element-binding proteins 1-) dependent responsive pathway which plays a central role among these transcriptional regulatory routes has been extensively studied and well characterized [15]. The CBF/DREB factors including CBF1/DREB1B, CBF2/DREB1C, and CBF3/DREB1A belong to the AP2/ERF (APETALA 2/ethylene-responsive factor) family, which can recognize and bind to the CRT/DRE (C-repeat/dehydration response element) cis-acting DNA regulatory element in the promoter regions of many cold-responsive (COR) genes [16, 17]. The COR genes with the ABRE (ABA-responsive element) or DRE/CRT element in their promoters decide to respond to the cold stress in the ABA-dependent or ABA-independent pathway [6, 18]. Recently, Kim et al. [19] revealed that two inducers of CBF expression (ICE1 and ICE2) can directly activate CBF1, CBF2, and CBF3 expression under low-temperature treatment and proposed a unified ICE-CBF cold acclimation pathway. Combining with the negative regulation of CBF2 to the transcription levels of *CBF1* and *CBF3* [20], ICE-CBF2 was considered as an attenuator to mediate cold signaling, which coordinated with HOS1-mediated attenuation of ICE activity at the protein level [19, 21]. In addition, extensive variation of CBFs and other genes in the cold-responsive transcriptome was gated by the circadian clock [22, 23], implying a crosstalk between circadian rhythm and cold signaling [24].

Strawberry is often subjected to cold stress in temperate regions with cold fall and winter temperatures and frequent spring frost events, when insulation measures are not strictly applied in protected cultivation [25, 26]. To provide the potential strategies in improving strawberry cold tolerance and give a glimpse into the understanding of the complex cold signaling pathways in plants, this study analyzed diverse regulatory networks that responded to cold stress and characterized multiple cold-responsive genes in strawberry leaves.

## 2. Results and Discussion

### 2.1. Genome-Wide Analysis of Transcriptional Response to Cold Stress

**Figure 1:**
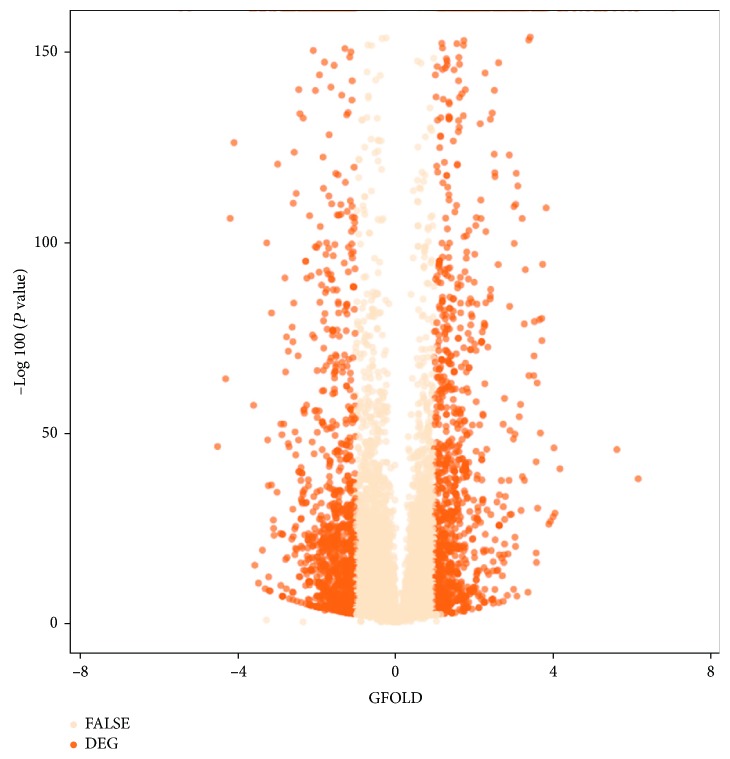
Volcano plot of genome-wide differentially expressed genes of T1 *vs*. CK. Genes with *P* value < 10^−5^ and GFOLD > 1 were indicated with colored dots and defined as robust differentially expressed ones (see Materials and Methods). DEG: differentially expressed gene; FALSE: filtered by the criteria above.

**Figure 2:**
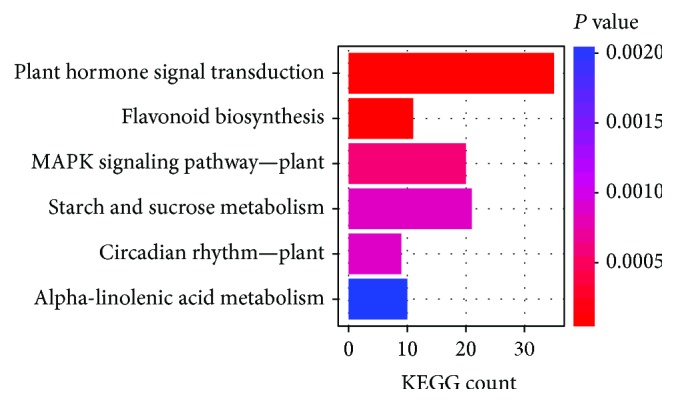
Functional enrichment analysis of differentially expressed genes. Pathways with adjusted *P* value < 0.05 were shown (see Materials and Methods).

Plants tightly coordinate environmental stimuli (e.g., cold stress) with gene expression and metabolism. To identify the cold-responsive gene expression profile in strawberry at the genome-wide level, we performed transcriptome analysis using RNA-Seq. Totally, there were 2397 differentially expressed genes (DEGs) under cold stress treatment (T1) *vs.* normal treatment (CK). Of these, 1180 DEGs were upregulated, while 1217 DEGs were downregulated (Figure 1, Table S1). This gene set number was quite comparable to a previous study as revealed in *Arabidopsis* [27], suggesting that cold stress triggered an extensive transcriptional reorganization in *Fragaria×ananassa* as that in *Arabidopsis*. It has been demonstrated that the amount of DEGs is linked with the exposure time at low temperature [28, 29]. Generally, the number of cold-responsive transcripts that increased to a maximum in a short term and short exposure to cold stress is certainly adequate if the purpose is to assess gene expression only [30, 31]. Thereby, this study applied the short-term stress strategy. However, this is not enough to provide deep insight into the physiological and metabolic aspects. A comprehensive consideration should be taken from the molecular, agronomic, or physiology perspective to bridge the knowledge gaps between short- and long-term effects of the genes and their products [32]. It has been documented that DEGs were distributed in various cold response pathways. Functional enrichment analysis showed that six pathways of plant hormone signal transduction, flavonoid biosynthesis, mitogen-activated protein kinase (MAPK) signaling, starch and sucrose metabolism, circadian rhythm, and alpha-linolenic acid metabolism were significantly (adjusted *P* value < 0.05) overrepresented in the DEGs (Figure 2, Table S2), indicating that those genes pronounced in the enriched pathways may play the pivotal roles in response to cold stress in strawberry.

### 2.2. Hormone Signaling Involved in Cold Response

**Figure 3:**
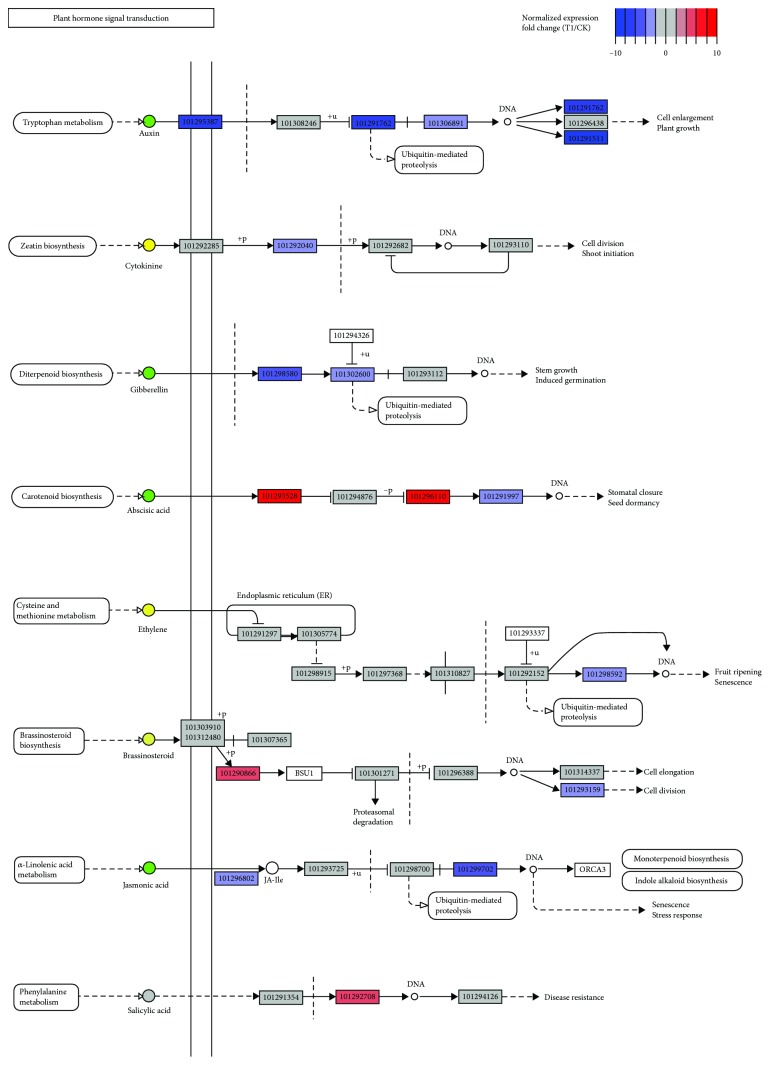
Hormone signaling pathways mapped with relative expression levels (T1 *vs*. CK). Gene ID of *F. vesca* is indicated at the corresponding gene node if there is.

As shown above, hormone signaling had the most significant change (*P* value = 2.79 × 10^−5^) during the cold stress process (Table S2). Many genes indeed displayed altered expression levels in several hormone-mediated branches, such as abscisic acid (ABA), auxin (IAA), cytokinin (CK), gibberellin (GA), ethylene (ET), brassinosteroid (BR), jasmonic acid (JA), and salicylic acid (SA) (Figure 3). These hormones play important roles in mediating plant defense response against abiotic and biotic stresses by amplifying the initial stress signal and initiating a second round of signaling [1, 33]. In recent years, melatonin has also been demonstrated to regulate key gene expression against stressors in abiotic and biotic stress conditions [34]. During recovery in wheat, foliar melatonin application could enhance the cold priming-induced tolerance to subsequent low-temperature stress [35]. Obviously, most of the hormone signaling pathways in strawberry leaves exposed to cold stress were inhibited, particularly for auxin, gibberellin, and jasmonic acid (Figure 3), which generally are obligated to cell elongation, division, cycle, and growth [36]. This is consistent with the well-documented knowledge that a remarkable growth reduction often occurs under cold stress [37]. In hormonal crosstalk that regulates cold stress-mediated growth and development of plants, auxin transport is one of the common targets, which changes the intracellular auxin gradient [7, 38]. Moreover, some components of the cold signaling pathway, like NUP160 and SIZ1, were demonstrated to link to auxin signaling [39–41]. More recently, the auxin-sensitive repressors (Aux/IAA) were proposed as hubs to integrate diverse environmental signals. Two Aux/IAA genes, *IAA5* and *IAA19*, were directly regulated by CBF1 and DREB2A in response to abiotic stress [42]. The auxin signaling severely hindered after low-temperature treatment in this study indicated its important role in cold response. Previous studies showed that GA and JA can cooperatively regulate diverse aspects of plant growth, development, and defense through DELLA and JAZ proteins [43, 44] and JA prioritizes defense over growth by interfering with GA signaling cascade [44]. In contrast, ABA and salicylic acid signaling branches were stimulated (Figure 3). Under low temperature or other elicitors, plants activate expression of downstream genes with cis-acting element ABRE and CRT/DRE in ABA-independent and ABA-dependent pathways. A transient accumulation of endogenous ABA is a typical physiological change in plants during cold stress, which contributes to cold tolerance [45], and the application of exogenous ABA enhanced cold resistance by elevating carbon isotopic fractionation, maintaining cell membrane stability, and optimizing photosystem II process [46]. Besides, ABA signal can integrate into sugar and reactive oxygen species signaling pathways to regulate plant cold tolerance and leaf senescence [6]. As shown, differentially expressed genes were enriched in the MAPK pathway and ABA, ethylene, and jasmonic acid routes that showed significantly impacted expression levels of genes in connection with abiotic stress defense (Figure S1). The transduction of second messengers and hormone signals by mitogen-activated protein kinases (MAPKs) in plants regulates gene expression to facilitate adaptation and survival in the face of diverse stresses [47, 48]. However, the precise mechanisms responsible for cold defense have yet to be deciphered.

### 2.3. Sugar Metabolism in Response to Cold Stress

**Figure 4:**
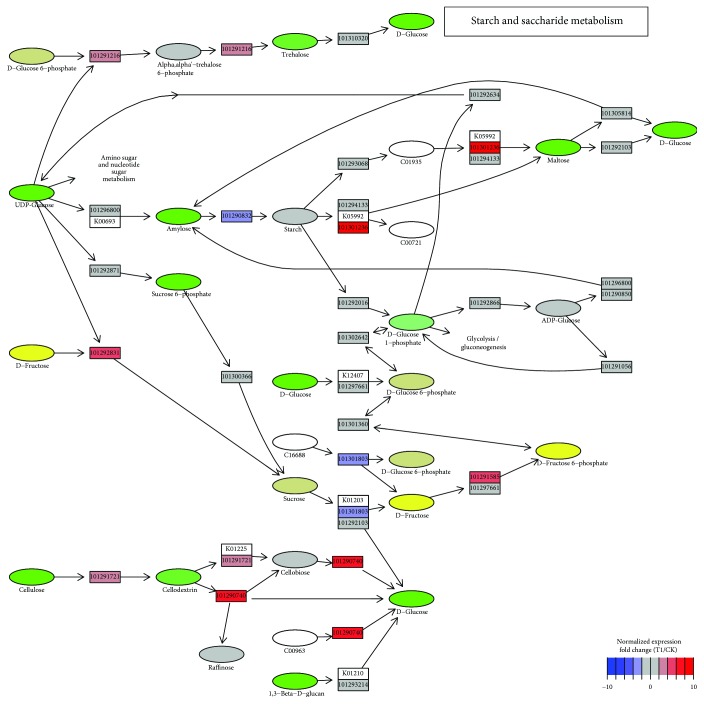
Starch and saccharide metabolism mapped with relative expression levels (T1 *vs*. CK). Gene ID of *F. vesca* is indicated at the corresponding gene node if there is.

Notably, the sugar metabolism network changed significantly in acclimation to cold stress (Figure 2). In addition to their essential roles as energy sources, carbon precursors, substrates for polymers, and storage and transport compounds, sugars can act as signals to regulate gene expression related to plant growth, development, metabolism, and stress resistance [4, 49]. As is well known, soluble sugars (saccharose, raffinose, stachyose, and trehalose), sugar alcohols (sorbitol, ribitol, and inositol) can be cryoprotectant molecules to save the cellular metabolism by protecting the integrity of membranes and cellular organelles in response to cold stress [50]. A remarkable trend could be observed that polysaccharides were degraded to soluble, simple sugars (Figure 4). For example, starch synthesis was inhibited, while its deconstruction to maltose/glucose was activated (Figure 4). The same metabolism flux also occurred in cellodextrin decomposition, which might be hydrolyzed/catabolized into glucose/raffinose by glycosidase (gene id 101290740) (Figure 4). These simple, easily metabolizable sugars did not likely undergo cellular respiration, as glycolysis, the pentose phosphate pathway, and the citrate cycle were not impacted (Figures S2-S4). Increased soluble sugars ensure a robust adaptation to future freezing stress [51]. Trehalose is believed to play a protective role against different abiotic stressful cues, and plants exhibit enhanced tolerance as a result of the trehalose biosynthesis gene overexpression [52].

Intriguingly, trehalose metabolism-related genes showed comparable expression levels to the CK (Figure 4), suggesting that trehalose was not regulated in the same manner as glucose/disaccharide, indicating its complex regulatory role during cold acclimation in plants [53].

### 2.4. Circadian Clock in Response to Cold Stimulus

**Figure 5:**
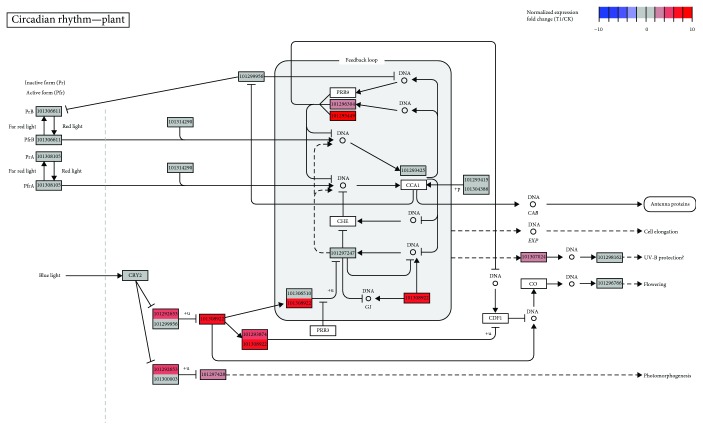
Circadian clock pathway mapped with relative expression levels (T1 *vs*. CK). Gene ID of *F. vesca* is indicated at the corresponding gene node if there is.

Circadian clock is an endogenous 24 h timekeeper that allows plant to anticipate the daily changes in the environment by adjusting biological activities and coordinates the responses to environmental stresses [24]. It is widely accepted that circadian rhythm is involved in cold acclimation in *Arabidopsis* and disruption of the circadian clock has indelible responsibility for extensive variation in the cold-responsive transcriptome [22, 23, 39]. Two major components in regulation of the circadian clock are circadian clock-associated 1 (*CCA1*) and late elongated hypocotyl (*LHY*), two MYB transcription factors that have been identified to positively regulate the CBF-dependent cold responsive pathway [39]. In strawberry, the ortholog of both CCA1 and LHY is a LHY-like protein (gene id 101293425; Figure 5). The expression level of the *LHY-like* gene was slightly repressed by cold stimulus (Table S1), suggesting a probable different regulatory network of the *LHY* component associated with the CBF pathway. Two additional elements of the circadian clock, *GIGANTEA* (*GI*) (gene id 101308922) and two-component response regulator-like PRR95 (gene id 101295449), were dramatically upregulated (approximately 60- to 70-fold; Figure 5). The *GI* gene previously has been identified as a circadian clock component involving flowering, phytochrome B signaling, and carbohydrate metabolism [54, 55]. Its role in mediating the cold acclimation in *Arabidopsis* has been investigated, possibly via a CBF-independent fashion [56]. Cold stress could enhance the GI expression, and then GI activates the production of soluble sugar, which is conducive to cold adaptation [57].

In our study, the *GI-like* gene was one of the most upregulated genes in response to cold stress (Table S1), indicating its important role during cold acclimation in strawberry. In addition, the two-component response regulator-like *PRR95* was also induced by cold stress (Figure 5 and Table S1). Although evidence has been found in maize that *PRR95* was sensitively regulated by cold temperature [58], its role in contribution to cold adaptation is under elucidation.

### 2.5. Flavonoid and *α*-Linolenic Acid Metabolism in Cold Response

**Figure 6:**
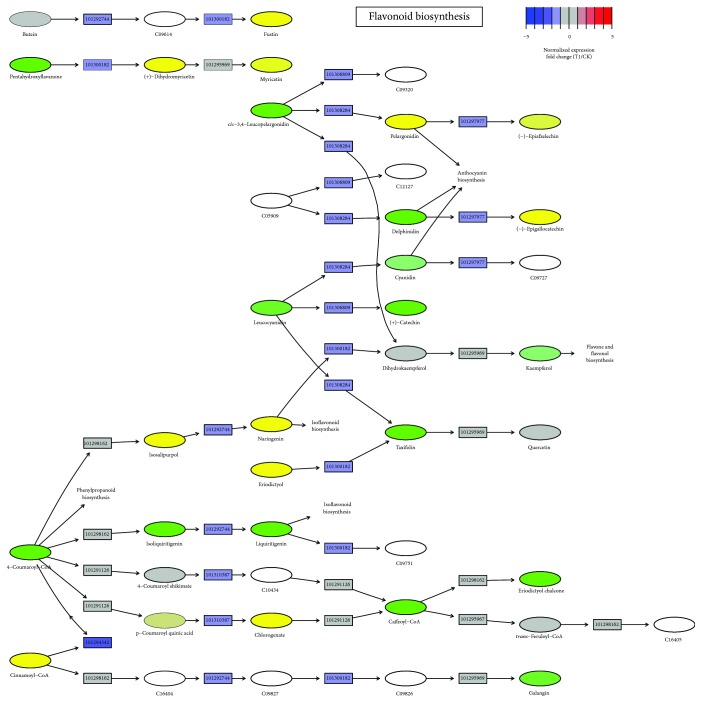
Flavonoid biosynthesis pathways mapped with relative expression levels (T1 *vs*. CK). Gene ID of *F. vesca* is indicated at the corresponding gene node if there is.

**Figure 7:**
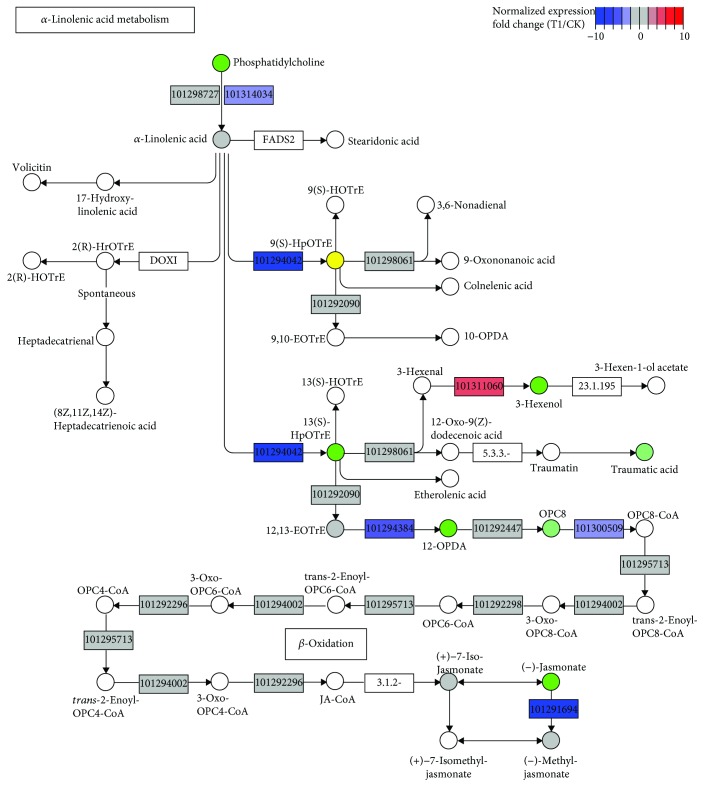
*α*-Linolenic acid metabolism pathways mapped with relative expression levels (T1 *vs*. CK). Gene ID of *F. vesca* is indicated at the corresponding gene node if there is.

Intriguingly, flavonoid and *α*-linolenic acid synthesis pathways were inactivated under cold stress as many genes were downregulated (Figures 6 and 7). It has been extensively reported that the induction of transcriptomic modifications directed towards the increase of flavonoid biosynthesis can increase resistance to cold stress and protect the plant from reactive oxygen species (ROS), especially those reactions involved in anthocyanin biosynthesis [59–61]. Moreover, flavonoids have been considered to modulate auxin transport in dependence on their quality by direct or indirect interactions with cellular transport and regulatory mechanisms and then control the plant growth and development [62–64]. Thus, the inhibition of flavonoid biosynthesis could be clearly biased with respect to affect the auxin transport in response to cold stress in strawberry. In addition, the reduction of secondary metabolism may benefit for energy economy, thus favoring plant overcoming the cold environment. Certain stimuli activate phospholipases to release *α*-linolenic acid (18 : 3) from membrane lipids, and then *α*-linolenic acid is converted to jasmonic acid (JA) by a series of enzymatic reactions [65, 66]. The data showed that the transcript levels of genes involving this pathway were significantly downregulated, which was consistent with the inhibition of jasmonic acid (JA) signaling, further emphasizing the important role of JA in response to cold stress. It has been documented that JA plays a positive role in improving plant cold tolerance by regulating the ICE-CBF cascade or ICE-CBF independent pathway [67]. Furthermore, JA is known to form crosstalk with other major phytohormones in response to cold stress [68].

### 2.6. Response of Cold Stress-Involved Components

**Figure 8:**
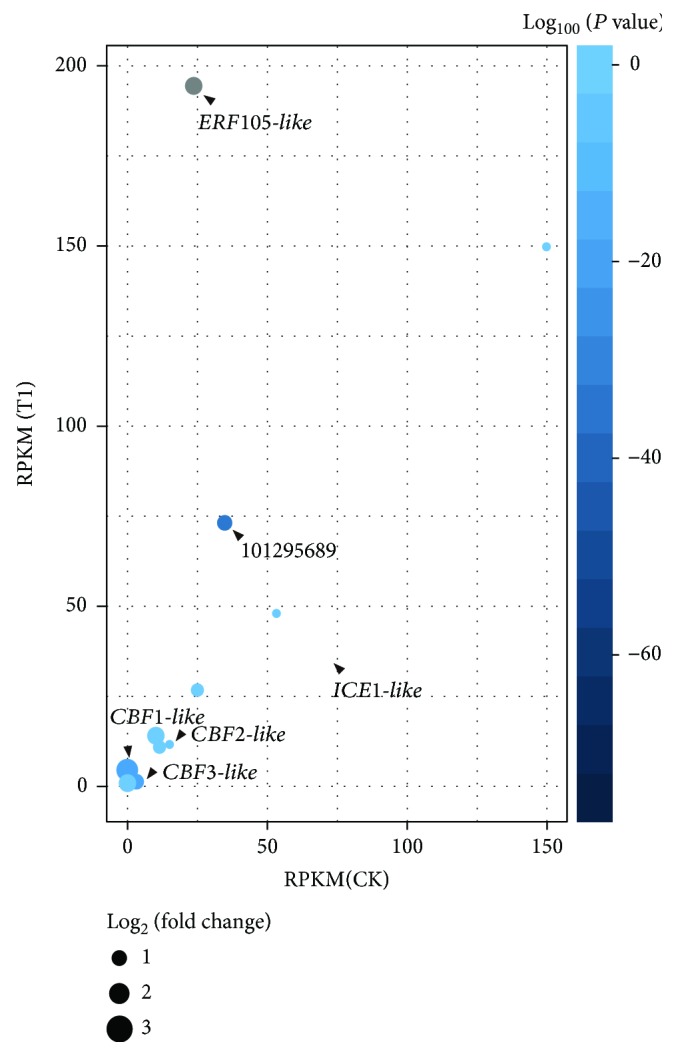
Transcriptional response of cold stress-involved components. Gene RPKM values of CK and T1 were plotted within the *x*-axis and *y*-axis, respectively. CBF-like genes and potential regulators in *F. vesca* were indicated by arrows.

The C-repeat binding factor (CBF) pathway is the best-documented regulatory pathway with a role in cold tolerance in *Arabidopsis* [29, 69]. The *CBF1-like* gene was indeed significantly upregulated in cold condition (Table S3), suggesting a role involved in cold adaptation in strawberry. However, all *CBF-like* genes were not highly transcribed (e.g., RPKM < 20) in cold stress condition as revealed in *Arabidopsis* (Figure 8 and Table S3) [27, 29]. This is in line with the expression of the *CBF* inducer *ICE1* (inducer of C-repeat binding factor expression 1) since *ICE1* was slightly downregulated, rather than induced by cold stimulus (Figure 8). Nevertheless, a recently identified transcription factor *ERF105-like* (gene id 101295082) was dramatically induced under cold stress condition (Figure 8), indicating its regulatory importance for cold acclimation in strawberry leaves. Whether *ERF105* is capable to function in combination with the CBF pathway needs to be investigated [70]. Moreover, some other ERF genes were demonstrated to function in cold tolerance [71]. For instance, PtrERF109 of trifoliate orange (*Poncirus trifoliata*) directly bound to the promoter of a peroxidase- (POD-) encoding gene PtrPrx1 to modulate ROS homoeostasis, thus playing a positive role in response to cold stress [72].

Additionally, a putative cold shock gene (gene id 101295689) was also highly expressed with a significant fold change (fold change > 2 and *P* value < 8.95 × 10^−69^; Figure 8). Further deciphering cold shock protein would benefit the cold stress study in plants and offer informative knowledge regarding the regulatory architecture of cold response in plants.

### 2.7. Validation of Digital Expression Profiles by qRT-PCR

**Figure 9:**
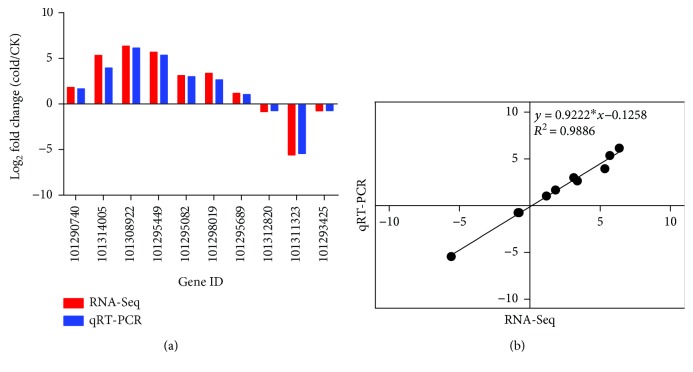
Verification of RNA-Seq results by qRT-PCR. (a) Comparison of the expression level of unique transcripts between RNA-Seq and qRT-PCR. Primers for qRT-PCR are listed in Supplementary Table 4. (b) Scatter diagram of log2 ratios from qRT-PCR and RNA-Seq results indicates the correlation between them. Gene ID of *F. vesca* is indicated at the corresponding gene node if there is.

To further confirm the reliability and accuracy of Illumina RNA-Seq expression profile data, we performed qRT-PCR assays on ten selected cold-responsive unique transcripts. As shown in Figure 9(a), all selected unique transcripts displayed expression profiles similar to those observed in RNA-Seq data. Moreover, the high correlation (*R* ^2^ = 0.98) described by a simple linear regression equation indicated the good consistency between the two analysis techniques (Figure 9(b)).

## 3. Conclusion

The mechanism of cold response in plants is very intricate, involving an array of physiological and biochemical modifications, multiple transcriptional regulatory pathway coordination, and cold-responsive gene expression alteration. Our study identified massive genes that significantly responded to cold stimuli in strawberry leaves, and these genes were significantly overrepresented in six pathways of plant hormone signal transduction, flavonoid biosynthesis, mitogen-activated protein kinase (MAPK) signaling, starch and sucrose metabolism, circadian rhythm, and alpha-linolenic acid metabolism. Most of the hormone signal pathways in strawberry leaves exposed to cold stress were inhibited, particularly for auxin, gibberellin, and jasmonic acid, while ABA and salicylic acid signaling branches were stimulated, indicating that a remarkable growth reduction can contribute to defend against cold stress in plants. A remarkable trend was observed that polysaccharides were degraded to soluble, simple sugars that could be cryoprotectant molecules. The significant orchestration of genes involving the circadian clock signaling suggested that disruption of the circadian clock had indelible responsibility for extensive variation in the cold-responsive transcriptome. Moreover, *GI*, *PRR95*, and *ERF105* were the attractive candidate genes for further study of cold stress in plants.

## 4. Materials and Methods

### 4.1. Plant Materials and Treatments

The strawberry seedlings were grown in plastic pots (120 mm × 100 mm) filled with a 1 : 1 (*v*/*v*) mixture of soil and perlite in the greenhouse of Sichuan Agricultural University. Temperature was set at 20/14°C (day/night), and relative humidity was 75%. Cold stress treatment was conducted on six-leaf-stage seedlings under natural condition. Strawberry seedlings were divided into two groups—the first group (at 20°C) was the control. As the cold stress treatment, another group was moved to an artificial intelligence growth chamber (4 ± 1°C, 88.8 *μ*mol·m^–2^·s^–1^·16 h·d^–1^, and 70 ± 5% relative humidity) for 4 days. The leaves were collected at 96 h after cold stress treatment and prepared in triplicate.

### 4.2. RNA Extraction, Sequencing, and Data Analysis

The leaves of strawberry were collected and immediately frozen in liquid nitrogen. Leaves were finely ground in liquid nitrogen, and ~100 mg ground powders were mixed with 1 mL TRIzol reagent (Invitrogen, Carlsbad, CA, USA), followed by adding 0.2 mL chloroform for protein denaturation. After centrifugation at 12000 g for 15 min, the supernatant was taken and then RNA was precipitated by adding 0.5 mL isopropanol. Total RNA was further purified by DNase I (RNeasy Mini Kit, QIAGEN, Hilden, Germany). RNA concentration and OD_260_/OD_280_ were measured with a Nanodrop 2000c (Thermo Scientific, Waltham, MA, USA), and RNA integrity was checked by agarose gel electrophoresis and an Agilent 2100 (Agilent Technologies, Santa Clara, CA, USA).

The qualified RNA with an OD_260_/OD_280_ > 1.8 and RIN (RAN integrity number) > 7.0 was prepared according to Shenzhen BGI (Shenzhen, China) and sequenced on an Illumina HiSeq 2500 platform (San Diego, CA, USA). Read mapping and counting using TopHat2 (version 2.0.12) [73] and HTSeq (version 0.6.0) [66] was performed with default parameters using the genome from the National Center for Biotechnology Information (NCBI) [74]. Mapped reads to exons were used to calculate normalized transcript abundance (defined by RPKM) [75] and to perform differential gene expression analysis by GFOLD (version 1.1.4) [76] and DEGseq (version 1.28.0) [77]. Genes with a DEGseq *P* value < 1 × 10^−4^ were considered to be statistically differentially expressed at a robust significance level. Because genes with ∣GFOLD∣ > 1 are empirically more likely to be of biological importance [76], a combined criterion of ∣GFOLD∣ > 1 and *P* < 1 × 10^−4^ was applied for genome-wide differential gene analysis [77]. RNA-Seq raw data are available at the SRA database under accession number PRJNA512251.

Functional enrichment analysis involves the statistical identification of a particular function category or expression subclass that is overrepresented in the whole gene collection. Total differentially expressed genes were submitted to the Kyoto Encyclopedia of Genes and Genomes (KEGG) [78], and the significantly enriched pathway (adjusted *P* value < 0.05) was estimated [79]. Enriched pathways were subsequently mapped by relative gene expression levels (*vs*. the CK sample) and visualized by the pathview [80].

### 4.3. Ortholog Searching

Putative orthologs of cold stress regulators (e.g., CBFs, ICE1, and ERF105) in *Arabidopsis thaliana* [29, 69, 81] were searched using BLASTP (identity > 50%, *E* value < 1 × 10^−10^, and coverage > 60%) against the genome-wide proteins of *F. vesca* (GCF_000184155.1).

### 4.4. Data Plotting

All figures were plotted on the R program platform (version 3.3.1) (http://www.r-project.org/).

## Data Availability

The NCBI SRA accession is PRJNA512251, and the release date is on 2020-01-27. My SRA records will be accessible with the following link after the indicated release date: https://www.ncbi.nlm.nih.gov/sra/PRJNA512251.

## Supplementary Materials

Supplementary 1 Figure S1: MAPK signaling pathway mapped with relative expression levels (T1 vs. CK).

Supplementary 2 Figure S2: glycolysis and gluconeogenesis pathways mapped with relative expression levels (T1 vs. CK).

Supplementary 3 Figure S3: pentose phosphate pathway mapped with relative 280 expression levels (T1 vs. CK).

Supplementary 4 Figure S4: citrate cycle mapped with relative expression levels (T1 vs. CK). Gene ID of F. vesca is indicated at the corresponding gene node if there is.

Supplementary 5 Table S1: genome-wide gene expression profiling and differential expression analysis.

Supplementary 6 Table S2: functional enrichment of differentially expressed genes.

Supplementary 7 Table S3: putative cold stress-involved components in strawberry.

Supplementary 8 Table S4: primer information of unique transcripts for the qRT-PCR analysis.

## References

[1] Peleg Z., Blumwald E. (2011). Hormone balance and abiotic stress tolerance in crop plants. *Current Opinion in Plant Biology*.

[2] Zhu J., Dong C.-H., Zhu J.-K. (2007). Interplay between cold-responsive gene regulation, metabolism and RNA processing during plant cold acclimation. *Current Opinion in Plant Biology*.

[3] Chinnusamy V., Zhu J., Zhu J.-K. (2007). Cold stress regulation of gene expression in plants. *Trends in Plant Science*.

[4] Janská A., Maršík P., Zelenková S., Ovesná J. (2010). Cold stress and acclimation–what is important for metabolic adjustment?. *Plant Biology*.

[5] Kumar S., Kaur G., Nayyar H. (2008). Exogenous application of abscisic acid improves cold tolerance in chickpea (*Cicer arietinum L.*). *Journal of Agronomy and Crop Science*.

[6] Xue-Xuan X., Hong-Bo S., Yuan-Yuan M. (2010). Biotechnological implications from abscisic acid (ABA) roles in cold stress and leaf senescence as an important signal for improving plant sustainable survival under abiotic-stressed conditions. *Critical Reviews in Biotechnology*.

[7] Rahman A. (2013). Auxin: a regulator of cold stress response. *Physiologia Plantarum*.

[8] Colebrook E. H., Thomas S. G., Phillips A. L., Hedden P. (2014). The role of gibberellin signalling in plant responses to abiotic stress. *Journal of Experimental Biology*.

[9] Fariduddin Q., Yusuf M., Ahmad I., Ahmad A. (2014). Brassinosteroids and their role in response of plants to abiotic stresses. *Biologia Plantarum*.

[10] Kazan K. (2015). Diverse roles of jasmonates and ethylene in abiotic stress tolerance. *Trends in Plant Science*.

[11] Miura K., Tada Y. (2014). Regulation of water, salinity, and cold stress responses by salicylic acid. *Frontiers in Plant Science*.

[12] Dong C.-J., Wang X.-L., Shang Q.-M. (2011). Salicylic acid regulates sugar metabolism that confers tolerance to salinity stress in cucumber seedlings. *Scientia Horticulturae*.

[13] Ljung K., Nemhauser J. L., Perata P. (2015). New mechanistic links between sugar and hormone signalling networks. *Current Opinion in Plant Biology*.

[14] Pirzadah T. B., Malik B., Rehman R. U., Hakeem K. R., Qureshi M. I., Hakeem K., Rehman R., Tahir I. (2014). Signaling in response to cold stress. *Plant signaling: Understanding the molecular crosstalk*.

[15] Shi Y., Ding Y., Yang S. (2018). Molecular regulation of CBF signaling in cold acclimation. *Trends in Plant Science*.

[16] Medina J., Bargues M., Terol J., Pérez-Alonso M., Salinas J. (1999). The Arabidopsis *CBF* gene family is composed of three genes encoding AP2 domain-containing proteins whose expression is regulated by low temperature but not by abscisic acid or dehydration. *Plant Physiology*.

[17] Medina J., Catalá R., Salinas J. (2011). The CBFs: three Arabidopsis transcription factors to cold acclimate. *Plant Science*.

[18] Zhang Q., Jiang N., Wang G.-L., Hong Y., Wang Z. (2013). Advances in understanding cold sensing and the cold-responsive network in rice. *Advances in Crop Science and Technology*.

[19] Kim Y. S., Lee M., Lee J.-H., Lee H. J., Park C. M. (2015). The unified ICE–CBF pathway provides a transcriptional feedback control of freezing tolerance during cold acclimation in Arabidopsis. *Plant Molecular Biology*.

[20] Novillo F., Alonso J. M., Ecker J. R., Salinas J. (2004). CBF2/DREB1C is a negative regulator of CBF1/DREB1B and CBF3/DREB1A expression and plays a central role in stress tolerance in Arabidopsis. *Proceedings of the National Academy of Sciences*.

[21] Dong C.-H., Agarwal M., Zhang Y., Xie Q., Zhu J. K. (2006). The negative regulator of plant cold responses, HOS1, is a RING E3 ligase that mediates the ubiquitination and degradation of ICE1. *Proceedings of the National Academy of Sciences*.

[22] Bieniawska Z., Espinoza C., Schlereth A., Sulpice R., Hincha D. K., Hannah M. A. (2008). Disruption of the Arabidopsis circadian clock is responsible for extensive variation in the cold-responsive transcriptome. *Plant Physiology*.

[23] Fowler S. G., Cook D., Thomashow M. F. (2005). Low temperature induction of Arabidopsis CBF1, 2, and 3 is gated by the circadian clock. *Plant Physiology*.

[24] Seo P. J., Mas P. (2015). STRESSing the role of the plant circadian clock. *Trends in Plant Science*.

[25] Maughan T. L., Black B. L., Drost D. (2015). Critical temperature for sub-lethal cold injury of strawberry leaves. *Scientia Horticulturae*.

[26] Zhang Y., Li Y., He Y. (2018). Identification of NADPH oxidase family members associated with cold stress in strawberry. *FEBS Open Bio*.

[27] Park S., Lee C. M., Doherty C. J., Gilmour S. J., Kim Y. S., Thomashow M. F. (2015). Regulation of the Arabidopsis CBF regulon by a complex low-temperature regulatory network. *The Plant Journal*.

[28] An D., Yang J., Zhang P. (2012). Transcriptome profiling of low temperature-treated cassava apical shoots showed dynamic responses of tropical plant to cold stress. *BMC Genomics*.

[29] Thomashow M. F. (2010). Molecular basis of plant cold acclimation: insights gained from studying the CBF cold response pathway. *Plant Physiology*.

[30] Fowler S., Thomashow M. F. (2002). Arabidopsis transcriptome profiling indicates that multiple regulatory pathways are activated during cold acclimation in addition to the CBF cold response pathway. *The Plant Cell*.

[31] Ganeshan S., Vitamvas P., Fowler D. B., Chibbar R. N. (2008). Quantitative expression analysis of selected COR genes reveals their differential expression in leaf and crown tissues of wheat (Triticum aestivum L.) during an extended low temperature acclimation regimen. *Journal of Experimental Botany*.

[32] Sanghera G. S., Wani S. H., Hussain W., Singh N. B. (2011). Engineering cold stress tolerance in crop plants. *Current Genomics*.

[33] Verma V., Ravindran P., Kumar P. P. (2016). Plant hormone-mediated regulation of stress responses. *BMC Plant Biology*.

[34] Arnao M. B., Hernández-Ruiz J. (2018). Melatonin: a new plant hormone and/or a plant master regulator?. *Trends in Plant Science*.

[35] Sun L., Li X., Wang Z. (2018). Cold priming induced tolerance to subsequent low temperature stress is enhanced by melatonin application during recovery in wheat. *Molecules*.

[36] Chaiwanon J., Wang W., Zhu J.-Y., Oh E., Wang Z. Y. (2016). Information integration and communication in plant growth regulation. *Cell*.

[37] Zhu J.-K. (2016). Abiotic stress signaling and responses in plants. *Cell*.

[38] Shibasaki K., Uemura M., Tsurumi S., Rahman A. (2009). Auxin response in *Arabidopsis* under cold stress: underlying molecular mechanisms. *The Plant Cell*.

[39] Dong M. A., Farré E. M., Thomashow M. F. (2011). Circadian clock-associated 1 and late elongated hypocotyl regulate expression of the C-repeat binding factor (CBF) pathway in *Arabidopsis*. *Proceedings of the National Academy of Sciences of the United States of America*.

[40] Miura K., Jin J. B., Lee J. (2007). SIZ1-mediated sumoylation of ICE1 controls CBF3/DREB1A expression and freezing tolerance in Arabidopsis. *The Plant Cell*.

[41] Miura K., Lee J., Gong Q. (2011). *SIZ1* regulation of phosphate starvation-induced root architecture remodeling involves the control of auxin accumulation. *Plant Physiology*.

[42] Shani E., Salehin M., Zhang Y. (2017). Plant stress tolerance requires auxin-sensitive Aux/IAA transcriptional repressors. *Current Biology*.

[43] Qi T., Huang H., Wu D. (2014). *Arabidopsis* DELLA and JAZ proteins bind the WD-repeat/bHLH/MYB complex to modulate gibberellin and jasmonate signaling synergy. *The Plant Cell*.

[44] Yang D.-L., Yao J., Mei C.-S. (2012). Plant hormone jasmonate prioritizes defense over growth by interfering with gibberellin signaling cascade. *Proceedings of the National Academy of Sciences of the United States of America*.

[45] Chen H.-H., Li P. H., Brenner M. L. (1983). Involvement of abscisic acid in potato cold acclimation. *Plant Physiology*.

[46] Huang X., Shi H., Hu Z. (2017). ABA is involved in regulation of cold stress response in bermudagrass. *Frontiers in Plant Science*.

[47] De Zelicourt A., Colcombet J., Hirt H. (2016). The role of MAPK modules and ABA during abiotic stress signaling. *Trends in Plant Science*.

[48] Smékalová V., Doskočilová A., Komis G., Šamaj J. (2014). Crosstalk between secondary messengers, hormones and MAPK modules during abiotic stress signalling in plants. *Biotechnology Advances*.

[49] Smeekens S., Hellmann H. A. (2014). Sugar sensing and signaling in plants. *Frontiers in Plant Science*.

[50] Bhandari K., Nayyar H., Ahmad P., Wani M. (2014). Low Temperature Stress in Plants: An Overview of Roles of Cryoprotectants in Defense. *Physiological mechanisms and adaptation strategies in plants under changing environment*.

[51] Guy C., Kaplan F., Kopka J., Selbig J., Hincha D. K. (2008). Metabolomics of temperature stress. *Physiologia Plantarum*.

[52] Kosar F., Akram N. A., Sadiq M., Al-Qurainy F., Ashraf M. (2019). Trehalose: a key organic osmolyte effectively involved in plant abiotic stress tolerance. *Journal of Plant Growth Regulation*.

[53] Fernandez O., Béthencourt L., Quero A., Sangwan R. S., Clément C. (2010). Trehalose and plant stress responses: friend or foe?. *Trends in Plant Science*.

[54] Dalchau N., Baek S. J., Briggs H. M. (2011). The circadian oscillator gene GIGANTEA mediates a long-term response of the Arabidopsis thaliana circadian clock to sucrose. *Proceedings of the National Academy of Sciences*.

[55] Fowler S., Lee K., Onouchi H. (1999). GIGANTEA: a circadian clock-controlled gene that regulates photoperiodic flowering in Arabidopsis and encodes a protein with several possible membrane-spanning domains. *The EMBO Journal*.

[56] Cao S., Ye M., Jiang S. (2005). Involvement of GIGANTEA gene in the regulation of the cold stress response in Arabidopsis. *Plant Cell Reports*.

[57] Gil K. E., Park C. M. (2019). Thermal adaptation and plasticity of the plant circadian clock. *New Phytologist*.

[58] Sobkowiak A., Jończyk M., Jarochowska E. (2014). Genome-wide transcriptomic analysis of response to low temperature reveals candidate genes determining divergent cold-sensitivity of maize inbred lines. *Plant Molecular Biology*.

[59] Butelli E., Licciardello C., Zhang Y. (2012). Retrotransposons control fruit-specific, cold-dependent accumulation of anthocyanins in blood oranges. *The Plant Cell*.

[60] Crifò T., Puglisi I., Petrone G., Recupero G. R., Lo Piero A. R. (2011). Expression analysis in response to low temperature stress in blood oranges: implication of the flavonoid biosynthetic pathway. *Gene*.

[61] Xie X. B., Li S., Zhang R. F. (2012). The bHLH transcription factor MdbHLH3 promotes anthocyanin accumulation and fruit colouration in response to low temperature in apples. *Plant, Cell & Environment*.

[62] Buer C. S., Imin N., Djordjevic M. A. (2010). Flavonoids: new roles for old molecules. *Journal of Integrative Plant Biology*.

[63] Peer W. A., Murphy A. S. (2007). Flavonoids and auxin transport: modulators or regulators?. *Trends in Plant Science*.

[64] Santelia D., Henrichs S., Vincenzetti V. (2008). Flavonoids redirect PIN-mediated polar auxin fluxes during root gravitropic responses. *Journal of Biological Chemistry*.

[65] Cheong J.-J., Do Choi Y. (2003). Methyl jasmonate as a vital substance in plants. *Trends in Genetics*.

[66] Dar T. A., Uddin M., Khan M. M. A., Hakeem K. R., Jaleel H. (2015). Jasmonates counter plant stress: a review. *Environmental and Experimental Botany*.

[67] Hu Y., Jiang Y., Han X., Wang H., Pan J., Yu D. (2017). Jasmonate regulates leaf senescence and tolerance to cold stress: crosstalk with other phytohormones. *Journal of Experimental Botany*.

[68] Per T. S., Khan M. I. R., Anjum N. A., Masood A., Hussain S. J., Khan N. A. (2018). Jasmonates in plants under abiotic stresses: crosstalk with other phytohormones matters. *Environmental and Experimental Botany*.

[69] Liu Q., Kasuga M., Sakuma Y. (1998). Two transcription factors, DREB1 and DREB2, with an EREBP/AP2 DNA binding domain separate two cellular signal transduction pathways in drought-and low-temperature-responsive gene expression, respectively, in Arabidopsis. *The Plant Cell*.

[70] Bolt S., Zuther E., Zintl S., Hincha D. K., Schmülling T. (2017). *ERF105* is a transcription factor gene of *Arabidopsis thaliana* required for freezing tolerance and cold acclimation. *Plant, Cell & Environment*.

[71] Klay I., Gouia S., Liu M. (2018). Ethylene response factors (ERF) are differentially regulated by different abiotic stress types in tomato plants. *Plant Science*.

[72] Wang M., Dai W., Du J., Ming R., Dahro B., Liu J.-H. (2018). ERF109 of trifoliate orange (*Poncirus trifoliata* (L.) Raf.) contributes to cold tolerance by directly regulating expression of *Prx1* involved in antioxidative process. *Plant Biotechnology Jjournal*.

[73] Love M. I., Huber W., Anders S. (2014). Moderated estimation of fold change and dispersion for RNA-seq data with DESeq2. *Genome Biology*.

[74] Anders S., Pyl P. T., Huber W. (2015). HTSeq—a Python framework to work with high-throughput sequencing data. *Bioinformatics*.

[75] Shulaev V., Sargent D. J., Crowhurst R. N. (2011). The genome of woodland strawberry (Fragaria vesca). *Nature Genetics*.

[76] Mortazavi A., Williams B. A., McCue K., Schaeffer L., Wold B. (2008). Mapping and quantifying mammalian transcriptomes by RNA-Seq. *Nature Methods*.

[77] Feng J., Meyer C. A., Wang Q., Liu J. S., Shirley Liu X., Zhang Y. (2012). GFOLD: a generalized fold change for ranking differentially expressed genes from RNA-seq data. *Bioinformatics*.

[78] Wang L., Feng Z., Wang X., Wang X., Zhang X. (2010). DEGseq: an R package for identifying differentially expressed genes from RNA-seq data. *Bioinformatics*.

[79] Kanehisa M., Sato Y., Kawashima M., Furumichi M., Tanabe M. (2016). KEGG as a reference resource for gene and protein annotation. *Nucleic Acids Research*.

[80] Yu G., Wang L.-G., Han Y., He Q. Y. (2012). clusterProfiler: an R package for comparing biological themes among gene clusters. *Omics*.

[81] Chinnusamy V., Ohta M., Kanrar S. (2003). ICE1: a regulator of cold-induced transcriptome and freezing tolerance in Arabidopsis. *Genes & Development*.

