# Supplementary material for: A Transcriptomic Analysis Reveals Diverse Regulatory Networks That Respond to Cold Stress in Strawberry (Fragaria×ananassa)
Source: Int J Genomics. 2019 Aug 5;2019:7106092. doi: 10.1155/2019/7106092 (PMC6701341; doi:10.1155/2019/7106092)
Supplement: Supplementary 3 — Figure S3: pentose phosphate pathway mapped with relative 280 expression levels (T1 vs. CK). [file 7106092.f3.pdf]

Figure S3

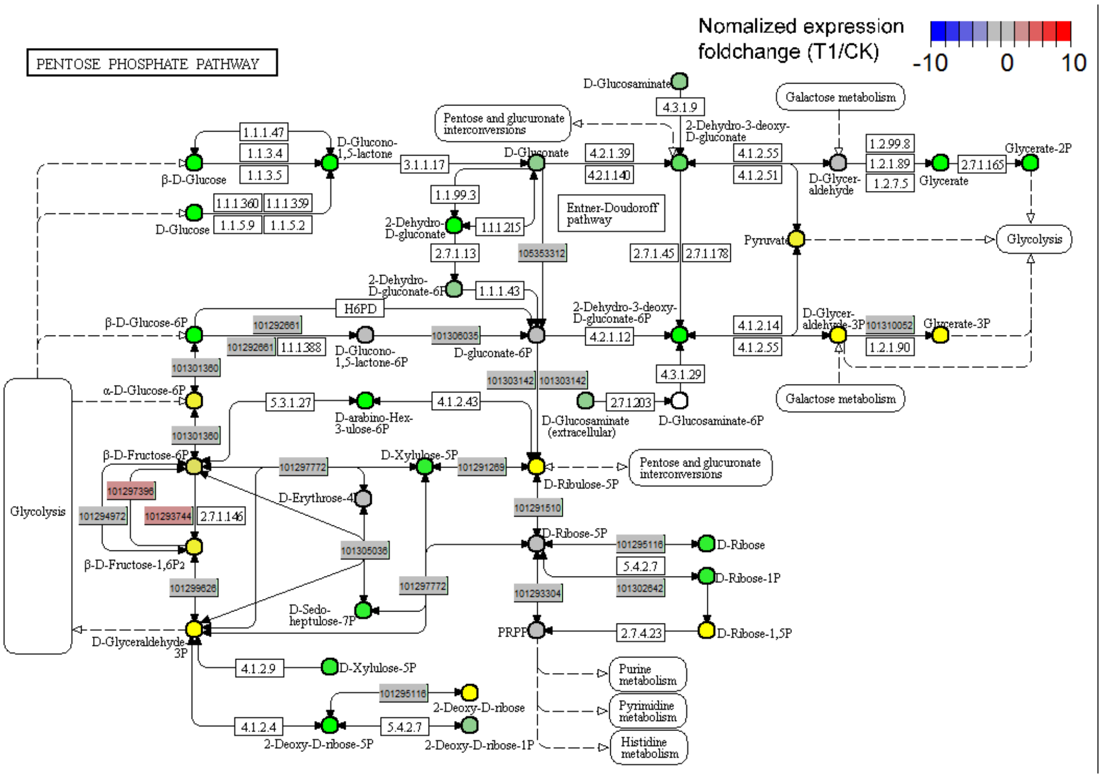

Figure S3. Pentose phosphate pathway mapped with relative expression levels (T1 *vs.* CK). Gene ID of *F. vesca* is indicated at the corresponding gene node if there is.
